# Systematic review and meta-analysis of physical rehabilitation on functional recovery in critically ill patients

**DOI:** 10.3389/fmed.2026.1877465

**Published:** 2026-08-18

**Authors:** Gang Wang, Xinghua Li, Linlin Fan, Li Lin, Zhiguo Yu, Qing Fang, Weihua Lu

**Affiliations:** Department of Emergency Medicine, General Hospital of Central Theatre Command, Wuhan, Hubei, China

**Keywords:** critically ill, ICU-acquired weakness, muscle strength, physical function, physical rehabilitation

## Abstract

**Purpose:**

Survivors of critical illness frequently experience persistent physical impairment, particularly ICU-acquired weakness (ICU-AW), which contributes to prolonged disability after intensive care. We conducted a systematic review and meta-analysis to evaluate the effects of physical rehabilitation on muscle strength, physical function, health-related quality of life, and clinical outcomes in critically ill adults.

**Methods:**

Four electronic databases were searched from inception to March 2026. Randomized controlled trials (RCTs) comparing physical rehabilitation with usual rehabilitation in adult critically ill patients were included. The primary outcome was muscle strength assessed by the Medical Research Council (MRC) sum score. Secondary outcomes included ICU-AW, physical function, health-related quality of life, duration of mechanical ventilation (MV), mortality, and ICU and hospital length of stay.

**Results:**

Forty RCTs involving 5,135 patients (2,554 physical rehabilitation; 2,581 usual rehabilitation) were included. Physical rehabilitation was associated with improved MRC sum score (SMD 0.26, 95% CI 0.05–0.46; *p* = 0.02; *I*^2^ = 70%), reduced incidence of ICU-AW (OR 0.62, 95% CI 0.43–0.87; *p* = 0.007; *I*^2^ = 34%), improved Barthel Index (SMD 0.62, 95% CI 0.21–1.03; *p* = 0.003; *I*^2^ = 85%) and SF-36 physical function (SMD 0.53, 95% CI 0.26–0.81; *p* < 0.001; *I*^2^ = 81%), shortened duration of MV (MD −1.32 days, 95% CI −1.98 to −0.65; *p* < 0.001; *I*^2^ = 66%) and ICU length of stay (MD −1.41 days, 95% CI −2.03 to −0.78; *p* < 0.001; *I*^2^ = 44%), but had no significant effects on mortality or hospital length of stay.

**Conclusion:**

Physical rehabilitation is associated with improved muscle strength and functional recovery, reduced ICU-AW, and shorter mechanical ventilation duration and ICU stay, but not reduced mortality. Further high-quality Multicenter trials are needed to define the optimal rehabilitation strategy.

**Systematic review registration:**

https://www.crd.york.ac.uk/PROSPERO/view/CRD42023462649, Identifier CRD42023462649.

## Introduction

Critically ill patients represent one of the most medically complex hospitalized population, and although advances in organ support have substantially improved survival, many survivors experience persistent physical, cognitive, and psychological impairment, collectively referred to as post-intensive care syndrome (PICS) (1, 2). Among these sequelae, physical disability is the most prevalent and has profound implications for long-term independence, health-related quality of life, and healthcare utilization (3, 4).

The pathophysiology of physical disability is dominated by rapid and extensive skeletal muscle wasting. Muscle mass loss may reach 30% within the first week of ICU admission (5), driven by systemic inflammation, prolonged immobility, sedation, and catabolic hormonal responses (3). This muscle wasting underpins ICU-acquired weakness (ICU-AW), which occurs in approximately 40–50% of mechanically ventilated patients and is independently associated with prolonged mechanical ventilation, increased hospital length of stay, impaired functional recovery, and reduced survival (6, 7).

Physical rehabilitation has therefore become a cornerstone of ICU recovery strategies (8). Contemporary rehabilitation programmes encompass a range of interventions, including early mobilization, progressive resistance exercise, in-bed cycle ergometry, neuromuscular electrical stimulation (NMES), and multidisciplinary rehabilitation involving physical and occupational therapy (9). Initiating rehabilitation early during critical illness may attenuate muscle wasting, preserve functional capacity, and accelerate recovery (10, 11). In selected studies, rehabilitation has also been combined with optimized protein provision to maximize muscle protein synthesis and enhance the anabolic response to exercise (12, 13).

Despite increasing adoption of ICU rehabilitation, its effects on patient-important outcomes remain uncertain. Previous systematic reviews were limited by relatively small sample sizes, heterogeneous rehabilitation strategies, and the absence of several recent large randomized controlled trials (RCTs) (14, 15). More recently, the TEAM trial (16) and the CYCLE trial (17) reported no significant improvements in hospital-free days and physical function, respectively, whereas Patel et al. (18) demonstrated a reduction in 1-year cognitive impairment following early physical and occupational therapy. Together, these landmark trials substantially expand and reshape the available evidence, warranting an updated synthesis.

Therefore, we conducted a systematic review and meta-analysis to evaluate the effects of different physical rehabilitation strategies, including mobilization-focused, exercise-based, and multimodal rehabilitation programmes, on muscle strength, physical function, health-related quality of life, and other clinically important outcomes in critically ill adults, with the Medical Research Council (MRC) sum score as the primary outcome.

## Methods

This systematic review and meta-analysis were conducted and reported in accordance with the Preferred Reporting Items for Systematic Reviews and Meta-Analyses (PRISMA 2020) guidelines (19). The protocol was registered in PROSPERO (CRD42023462649).

### Search strategy

Two investigators (GW and XL) independently searched PubMed, EMBASE, the Cochrane Central Register of Controlled Trials (CENTRAL), and SCOPUS from database inception to March 2026. The following keywords and MeSH terms were used: (“critical illness” OR “critically ill” OR “critical care” OR “intensive care” OR “intensive care unit” OR ICU OR “mechanical ventilation” OR “mechanically ventilated”) AND (“physical rehabilitation” OR rehabilitation OR “physical therapy” OR physiotherapy OR mobilization OR mobilization OR “early mobilization” OR “early mobilization” OR exercise OR “exercise therapy” OR “resistance training” OR “strength training” OR cycling OR “cycle ergometry” OR “neuromuscular electrical stimulation” OR NMES) AND (“randomized controlled trial” OR “randomised controlled trial” OR randomized OR randomized OR trial OR RCT). Additionally, reference lists of included studies and relevant systematic reviews were hand-searched.

### Selection criteria

#### Inclusion criteria

1. Population: Adult (≥18 years old) critically ill patients;
2. Intervention: Physical rehabilitation initiated at any time between ICU admission and hospital discharge. Eligible interventions included mobilization-focused, exercise-based, or multimodal rehabilitation programmes, encompassing passive or active mobilization, endurance exercise, resistance training, cycle ergometry, neuromuscular electrical stimulation (NMES), and physical and/or occupational therapy, irrespective of timing, intensity, frequency, or duration.
3. Comparison: Usual rehabilitation or standard rehabilitation.
4. Outcomes: Studies reporting at least one prespecified outcome of interest related to muscle strength, physical function, health-related quality of life, or other clinically important outcomes.
5. Study design: RCTs (including pilot and feasibility trials).

#### Exclusion criteria

Studies were excluded if they lacked a comparator group, did not report any prespecified outcome of interest, or evaluated rehabilitation initiated after hospital discharge.

### Data extraction

Data from included studies were extracted by two independent authors (GW and XL) and discrepancies were resolved through discussion with a senior third-person adjudicate (WL) until consensus was reached. The data extracted from the included studies was: the first author’s name, year of publication, the country where the study was from, number of participants, study population, timing, type and duration of physical rehabilitation, and relevant outcome measures. For studies reporting median (interquartile range) data, means and standard deviations were estimated using the Wan 2014 method to enable pooling in meta-analyses (20).

### Outcomes

The primary outcome was muscle strength assessed using the MRC sum score. Secondary outcomes included incidence of ICU-AW, measures of physical function (6-min walk distance [6MWD], handgrip strength, quadriceps strength, Barthel Index, Functional Status Score for the ICU [FSS-ICU], and Functional Independence Measure [FIM]), health-related quality of life (SF-36 physical function domain), duration of mechanical ventilation (MV), ICU length of stay, hospital length of stay, and overall mortality.

### Assessment of risk of bias

Two reviewers (GW and XL) independently assessed the risk of bias the Cochrane Risk of Bias tool version 2 (RoB 2) (21). The following five domains were evaluated: bias arising from the randomization process, bias due to deviations from intended interventions, bias due to missing outcome data, bias in the measurement of the outcome, and bias in the selection of the reported result. Risk-of-bias plots were generated using the robvis visualization tool (22).

### Statistical analysis

Meta-analyses were performed using Review Manager (RevMan) version 5.4 (The Nordic Cochrane Centre, Copenhagen, Denmark). Additional statistical analyses were conducted using R software (version 4.4.1) with the metafor package. For dichotomous outcomes, pooled effect estimates were expressed as odds ratios (ORs) with 95% confidence intervals (CIs). For continuous outcomes, mean differences (MDs) or standardized mean differences (SMDs) with 95% CIs were calculated, depending on the consistency of outcome measurements across studies. Statistical heterogeneity was assessed using the *I*^2^ statistic. As substantial clinical and methodological heterogeneity was anticipated owing to differences in rehabilitation strategies, intervention timing, intensity, and patient populations, a random-effects model was prespecified and applied to all meta-analyses regardless of the degree of statistical heterogeneity. Publication bias was assessed by visual inspection of funnel plots for outcomes including at least 10 studies and further evaluated using Egger’s regression test and Begg’s rank correlation test. For outcomes with substantial heterogeneity, leave-one-out sensitivity analyses, Baujat plots, and influence diagnostics were performed to identify influential studies and explore potential sources of heterogeneity. Statistical significance was defined as a two-sided *p* value < 0.05.

Certainty of evidence was assessed for all outcomes using the GRADE (Grading of Recommendations Assessment, Development and Evaluation) approach, considering five domains: risk of bias, inconsistency, indirectness, imprecision, and publication bias (23).

## Results

### Study selection

**Figure 1:**
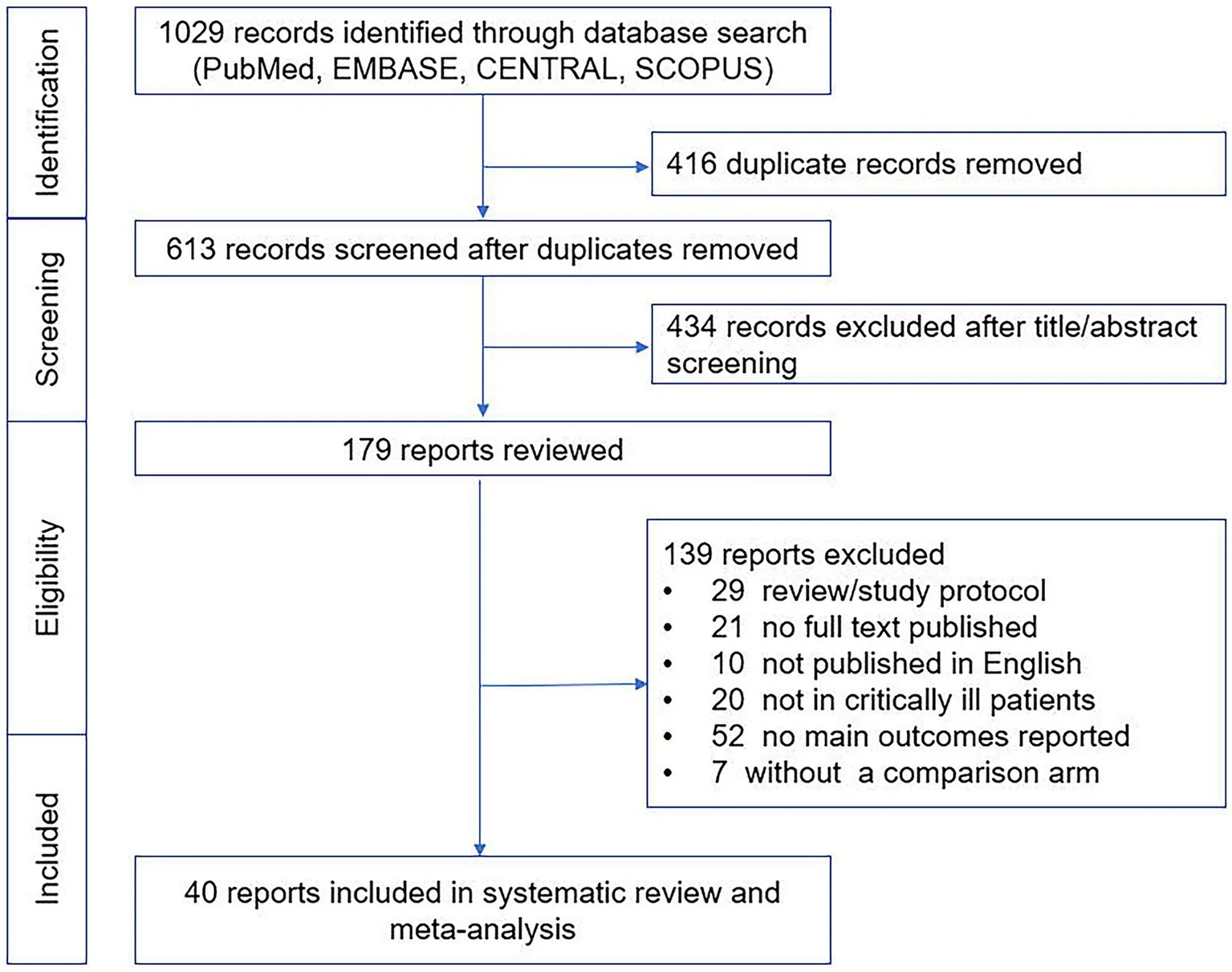
Flow diagram that summarizes the results of the literature search.

The initial database search identified 1,029 records. After removing 416 duplicates, 613 records were screened by title and abstract, of which 434 were excluded. A total of 179 full-text reports were assessed for eligibility. Following application of inclusion and exclusion criteria, 40 studies were included in the systematic review and meta-analysis (Figure 1) (16–18, 24–60).

### Study characteristics

A total of 40 randomized controlled trials involving 5,135 critically ill adults were included, comprising 2,554 patients allocated to physical rehabilitation and 2,581 to usual rehabilitation. The characteristics of the included studies are summarized in Table 1. Studies were conducted across North America, Europe, Asia, Australia, and New Zealand and enrolled heterogeneous ICU populations. The evaluated rehabilitation strategies included mobilization-focused, exercise-based, and multimodal rehabilitation programmes, incorporating interventions such as passive or active mobilization, cycle ergometry, resistance exercise, NMES, and physical and occupational therapy. Rehabilitation was initiated between 24 and 96 h after ICU admission in most studies. The median study duration was 28 days (range, 7–180 days). The largest included trials were the multinational TEAM trial (750 participants from 49 hospitals in six countries) (16) and the multicenter CYCLE trial (360 participants from 16 ICUs in three countries) (17).

**Table 1:** Characteristics of the randomized controlled trials included in the systematic review and meta-analysis.

Author, year, country	Population	Initiation timing	Intervention	control	Physical function, muscle strength or quality of life outcomes
Burtin, 2009, Belgium (24)	90 ICU patients with predicted ICU stay >7 days	Day 5	Exercise-based rehabilitation: cycle ergometry (20 min/session, 5 days/week; individualized intensity).	Physiotherapy and standardized mobility of upper and lower extremities ranging from passive to active.	6-min walk test (6MWT) at hospital discharge; quadriceps strength; Handgrip strength; Berg Balance Scale; Functional Ambulation Categories scale; SF-36 (physical function domain, PCS)
Schweickert, 2009, USA (25)	104 ICU patients on mechanical ventilation (MV) for <72 h and expected to continue for >24 h	The day of enrolment	Multimodal rehabilitation: daily progressive PT/OT from passive ROM to ADLs and ambulation.	Usual rehabilitation	ADL scores Barthel Index; Number of functionally independent ADLs; Distance walked without assistance; MRC score; Handgrip strength; duration of MV.
Chen, 2012, Taiwan (26)	27 patients with prolonged mechanical ventilation	Not mentioned	Mobilization-focused rehabilitation: endurance, strengthening and stretching (30–40 min/session).	Usual rehabilitation	physical functional status: Barthel index and the Functional Independence Measurement (FIM); duration of MV
Dantas, 2012, Brazil (27)	59 ICU patients on MV.	Not mentioned	Mobilization-focused rehabilitation: protocolized early mobilization twice daily.	Passive mobility of the four limbs 5 times a week and active assisted exercises.	Medical Research Council (MRC) score
Dong, 2014, China (28)	60 ICU patients with MV 48–72 h and with predicted MV > 7 days	48–72 h	Mobilization-focused rehabilitation: progressive out-of-bed mobility twice daily.	Usual rehabilitation	Time to first sit out of bed; duration of MV.
Kayambu, 2015, Australia (30)	50 ICU patients with sepsis and with MV > 48 h	within 48 h of the diagnosis of sepsis	Exercise-based rehabilitation: individualized exercise with EMS and progressive mobilization.	Usual rehabilitation (respiratory and functional mobility)	Acute Care Index of Function at ICU discharge; SF-36 at 6 months post-discharge; MRC score.
Walsh, 2015, Scotland (31)	240 ICU patients with MV > 48 h	After ICU discharge	Multimodal rehabilitation: intensified rehabilitation with goal setting, dietetic support and education.	Usual rehabilitation (a self-help ICU rehabilitation manual)	Rivermead Mobility Index (RMI) at 3 months; Handgrip strength; SF-12 PCS and MCS; 2-m TUG test; Hospital Anxiety and Depression Scale (HADS).
Yosef-Brauner, 2015, Israel (29)	18 ICU patients with MV ≥ 48 h and expected to remain ventilated > 96 h	Not mentioned	Exercise-based rehabilitation: intensive conventional physiotherapy twice daily.	Usual rehabilitation (conventional physiotherapy)	MRC score; Handgrip strength; Sitting balance
Hodgson, 2016, Australia (32)	50 ICU patients with MV > 48 h	Not mentioned	Mobilization-focused rehabilitation: early goal-directed mobility (up to 1 h/day).	Usual rehabilitation (standard care)	higher maximal level and duration of activity measured using ICU mobility Scale; Functional Status Score in ICU test (FSS-ICU); MRC score; Duration of MV; EQ-5D
Morris, 2016, USA (33)	300 ICU patients with MV	<48 h	Mobilization-focused rehabilitation: standardized progressive rehabilitation three sessions/day.	Usual rehabilitation	hospital length of stay; Short Physical Performance Battery (SPPB); SF-36 (physical function domain); Handgrip strength and handheld strength; Duration of MV
Schaller, 2016, Germany (34)	200 ICU patients with MV < 48 h and expected further MV > 24 h	<48 h	Multimodal rehabilitation: interprofessional goal-directed mobilization (SOMS-guided).	Usual rehabilitation	surgical ICU optimal mobilization score (SOMS) modified functional independence measure (FIM); MRC score; SF-36; Ventilator-free days.
Machado, 2017, Brasil (35)	38 ICU patients who were on MV	Median 2 days	Exercise-based rehabilitation: passive cycle ergometry (20 min, 5 days/week).	Conventional physical and respiratory therapy.	MRC score, Duration of MV.
Maffei, 2017, France (36)	40 ICU liver transplant recipients	48–72 h	Mobilization-focused rehabilitation: early progressive mobility twice daily.	Usual rehabilitation (physiotherapy with 1 session per day)	Time to first mobility milestones (sitting on the edge of the bed, sitting in the chair, walking and transit); Duration of MV.
Patsaki, 2017, Greece (37)	128 patients with MV > 72 h	Within 48 h of ICU discharge	Exercise-based rehabilitation: NMES plus individualized rehabilitation.	Usual rehabilitation	MRC score, handgrip strength, Functional Independence Measure (FIM).
Wright, 2018, UK (38)	308 ICU patients with MV ≥ 48 h	<72 h	Mobilization-focused rehabilitation: protocolized physiotherapy (90 min/day, 5 days/week).	Usual rehabilitation	SF-36 (physical function domain); modified Rivermead Mobility Index; 6MWT; FIM; Handgrip strength
Eggmann, 2018, Switzerland (39)	Mixed ICU patients on MV with ICU stay ≥72 h	<48 h	Exercise-based rehabilitation: cycling plus resistance training (up to 3 sessions/day).	Usual rehabilitation (early mobility, respiratory therapy and passive/active exercises)	6MWT and FIM; quadriceps strength; Handgrip strength; MRC score; TUG test; SF-36
Fossat, 2018, France (40)	314 ICU patients admitted to ICU < 72 h before randomization	<48 h	Exercise-based rehabilitation: cycle ergometry plus quadriceps NMES.	Usual rehabilitation (standardized early rehabilitation)	MRC score ICU Mobility Scale; Katz ADL; Barthel Index; SF-36
McWilliams, 2018, UK (42)	103 ICU patients with MV ≥ 5 days	>5 days	Mobilization-focused rehabilitation: structured individualized mobility programme with weekly goals.	Usual rehabilitation	Manchester Mobility score; Barthel index; handgrip strength; SF-36 (physical function); Duration of MV.
Verceles, 2018, USA (43)	32 ICU patients with prolonged MV for ≥14 days	>14 days	Mobilization-focused rehabilitation: patient-specific progressive mobility programme.	Usual rehabilitation (basic rehabilitation activities)	Handgrip strength; 6MWT; SPPB
Hickmann, 2018, Belgium (41)	19 ICU patients with septic shock <72 h	<48 h	Mobilization-focused rehabilitation: twice-daily cycling plus limb mobilization.	Usual rehabilitation (5 times per week, functional mobility)	regulation of protein degradation/− synthesis pathways during the first week; muscle fibre CSA; Exercise-induced muscle inflammation; Duration of MV.
Kho, 2019, Canada (44)	66 ICU patients with <4 d of MV and <7 d ICU stay	<72 h	Exercise-based rehabilitation: in-bed cycling plus usual physiotherapy.	Usual rehabilitation	Duration of MV; clinical frailty score (CFS)
Seo, 2019, Korea (56)	16 ICU patients with ICU stay ≥5 days	>5 d	Mobilization-focused rehabilitation: progressive mobility programme.	Usual rehabilitation	MRC-SS; FSS-ICU; SF-36
Berney, 2021, Australia (45)	162 ICU patients with MV ≥ 48 h and ICU stay ≥4 d	<72 h	Exercise-based rehabilitation: FES-assisted cycling (≥5 days/week).	Usual rehabilitation (respiratory and functional mobility)	quadriceps strength MRC score; handgrip strength; FSS-ICU; SPPB; 6MWT; Katz ADL; cross-sectional area of rectus femoris (RF-CSA); Duration of MV.
Nickels, 2020, Australia (46)	72 ICU patients with expected MV > 48 h	<96 h	Exercise-based rehabilitation: daily in-bed cycling plus usual physiotherapy.	Usual rehabilitation (respiratory and functional mobility)	RF-CSA at Day 10 RF and Vastus intermedius (VI) thickness; MRC score; Handgrip strength; FSS-ICU; 6MWT; ICU Mobility Scale; Duration of MV.
Schujmann, 2020, Brazil (49)	99 ICU patients	<48 h	Mobilization-focused rehabilitation: progressive mobility plus conventional therapy.	Conventional therapy involving active assists and active mobilization as well as bed positioning, bedside and armchair transfers and ambulation.	Barthel Index; handgrip strength; TUG test; Sit to stand test; 2-min walk test; ICU Mobility Score; duration of MV.
Nydahl, 2020, Germany (47)	274 ICU patients	Median 3 days	Mobilization-focused rehabilitation: goal-directed mobility using the ICU Mobility Scale.	Usual rehabilitation	Mobility level (ICU Mobility Scale), Functional Status Score for the ICU (FSS-ICU), Barthel Index, health-related quality of life (EQ-5D)
Dos Santos, 2020, Brazil (48)	144 ICU patients with MV < 72 h	< 72 h	Exercise-based rehabilitation: resistance exercise plus NMES twice daily.	Usual rehabilitation	Functional mobility, ICU Mobility Scale (IMS), Functional Status Score for the ICU (FSS-ICU), length of functional recovery
de Azevedo, 2021, Brazil (50)	181 patients on MV with expected ICU stay >3 days	Not mentioned	Multimodal rehabilitation: optimized nutrition plus cycle ergometry.	Usual rehabilitation (routine physiotherapy)	SF-36 PCS at 3 months and 6 months; incidence of ICU-AW; duration of MV.
Nakamura, 2021, Japan (51)	117 ICU patients	Not mentioned	Multimodal rehabilitation: high-protein nutrition plus individualized exercise.	Usual rehabilitation	femoral muscle volume change (%); FSS-ICU; Barthel index; EQ-5D; duration of MV.
Hodgson, 2022, International	750 MV patients (≥18 yr)	Within 72 h of ICU admission.	Mobilization-focused rehabilitation: high-dose goal-directed active mobilization.	Usual rehabilitation (standard ICU mobilization).	Days alive and out of hospital at D180 (primary); VFD at D28; 180-day mortality; Barthel ADL at D180; EQ-5D-5L; WHODAS 2.0; adverse events
Kagan, 2022, Israel (53)	41 ICU patients with MV > 48 h and expected further MV > 7 d	>48 h	Multimodal rehabilitation: cycle ergometry plus protein-enriched enteral nutrition.	Usual rehabilitation (conventional physiotherapy)	Duration of MV.
Verceles, 2023, USA (54)	39 ICU patients with MV ≥ 24 h	>24 h	Multimodal rehabilitation: NMES, high-protein supplementation and mobility training.	Usual rehabilitation	cross sectional area and volume of thigh and lower leg muscle; Duration of MV
Zhou, 2022, China (55)	150 patients with expected ICU stay ≥72 h	<24 h	Multimodal rehabilitation: early mobilization plus early nutrition.	Usual rehabilitation (standard care)	incidence of ICU-AW at ICU discharge; MRC score, Barthel Index, duration of MV
Yu, 2022, China (52)	102 ICU patients with MV for 1–2 days with ICU stay of at least 7 days	>48 h	Exercise-based rehabilitation: bedside leg ergometry.	Usual rehabilitation	MV-free days; length of ICU and hospital stay
Patel, 2023, USA (18)	200 functionally independent MV adults within first 96 h of MV	Within 96 h of MV initiation	Mobilization-focused rehabilitation: early PT/OT with progressive mobility.	Usual rehabilitation (standard ICU PT/OT as prescribed by treating team)	Cognitive impairment at 1 year (MoCA<26; primary); ICU-AW at discharge and 1 year; functional independence (FIM); SF-36 PCS; MV duration; ICU/hospital LOS
Kho, 2024, Canada/USA/Australia (17)	360 MV adults in medical-surgical ICU	Within 4 days of MV initiation.	Exercise-based rehabilitation: in-bed cycle ergometry plus usual physiotherapy.	Usual rehabilitation (usual physiotherapy)	PFIT-s at 3 days post-ICU discharge (primary); 6MWT; frailty (CFS); QoL; ICU-AW (MRC < 48); 90-day mortality; MV duration; ICU/hospital LOS
Yen, 2024, Taiwan (59)	65 ICU patients with moderate-to-severe TBI (GCS 6–13), on MV	Within 7 days of injury	Mobilization-focused rehabilitation: early progressive out-of-bed mobilization.	Usual rehabilitation (early progressive upright positioning)	Perme ICU Mobility Score (primary); FIM-motor; phase angle; skeletal muscle index; ICU LOS; MV duration
Yokobatake, 2025, Japan (60)	44 ICU patients aged ≥65 yr. with APACHE II > 20	Within 48 h of ICU admission	Exercise-based rehabilitation: NMES plus early mobilization.	Early mobilization alone (no NMES)	QIS at hospital discharge (primary); 6MWD; Barthel Index; MRC score; handgrip strength; ICU-AW incidence; ICU/hospital LOS
Guerra-Vega, 2025, Chile (58)	54 ICU MV patients	within 24 h of ICU admission	Exercise-based rehabilitation: medium−/low-frequency NMES plus standard physiotherapy.	Usual rehabilitation (standard physiotherapy)	MRC-SS; handgrip strength; Barthel Index; SF-36 (PCS and MCS); TUG test; FSS-ICU; ICU-AW incidence; hospital LOS; MV duration; ICU LOS
Cusack, 2025, UK (57)	46 intubated/ventilated ICU patients (<72 h MV, expected >48 h more) across 3 UK ICUs	Within 72 h of intubation	Mobilization-focused rehabilitation: protocolized early rehabilitation plus usual physiotherapy.	Usual rehabilitation (routine PT)	EQ-5D-5L; Duration of MV; ICU/hospital LOS.

6MWD, 6-min walk distance; 6MWT, 6-min walk test; ADL, activities of daily living; APACHE II, Acute Physiology and Chronic Health Evaluation II; Barthel Index, Barthel Activities of Daily Living Index; CFS, Clinical Frailty Scale; CSA, cross-sectional area; EQ-5D, EuroQol 5-Dimension questionnaire; FES, functional electrical stimulation; FIM, Functional Independence Measure; FSS-ICU, Functional Status Score for the Intensive Care Unit; GCS, Glasgow Coma Scale; HADS, Hospital Anxiety and Depression Scale; ICU, intensive care unit; ICU-AW, intensive care unit-acquired weakness; IMS, ICU Mobility Scale; LOS, length of stay; MCS, Mental Component Summary; MoCA, Montreal Cognitive Assessment; MRC, Medical Research Council sum score; MV, mechanical ventilation; NMES, neuromuscular electrical stimulation; OT, occupational therapy; PCS, Physical Component Summary; PFIT-s, Physical Function in Intensive Care Test–scored; PT, physical therapy; QIS, quadriceps isometric strength; RF, rectus femoris; RF-CSA, rectus femoris cross-sectional area; ROM, range of motion; SF-12, 12-Item Short Form Health Survey; SF-36, 36-Item Short Form Health Survey; SOMS, Surgical Optimal Mobilization Score; SPPB, Short Physical Performance Battery; TBI, traumatic brain injury; TUG, Timed Up and Go test; VFD, ventilator-free days; VI, vastus intermedius; WHODAS 2.0, World Health Organization Disability Assessment Schedule 2.0.

### Risk of Bias assessment

Detailed risk-of-bias assessments are presented in Supplementary Figures 1, 2. Overall, the methodological quality of the included studies was moderate to high. Most studies were judged to have a low risk of bias for randomization and deviations from intended interventions. As expected for rehabilitation trials, blinding of participants and treating therapists was generally not feasible but was not considered sufficient to indicate a high risk of bias in accordance with the RoB 2 guidance (21). Some concerns were primarily related to missing outcome data, outcome measurement, and selective reporting.

### Meta-analysis

#### Primary outcome: muscle strength (MRC sum score)

**Figure 2:**
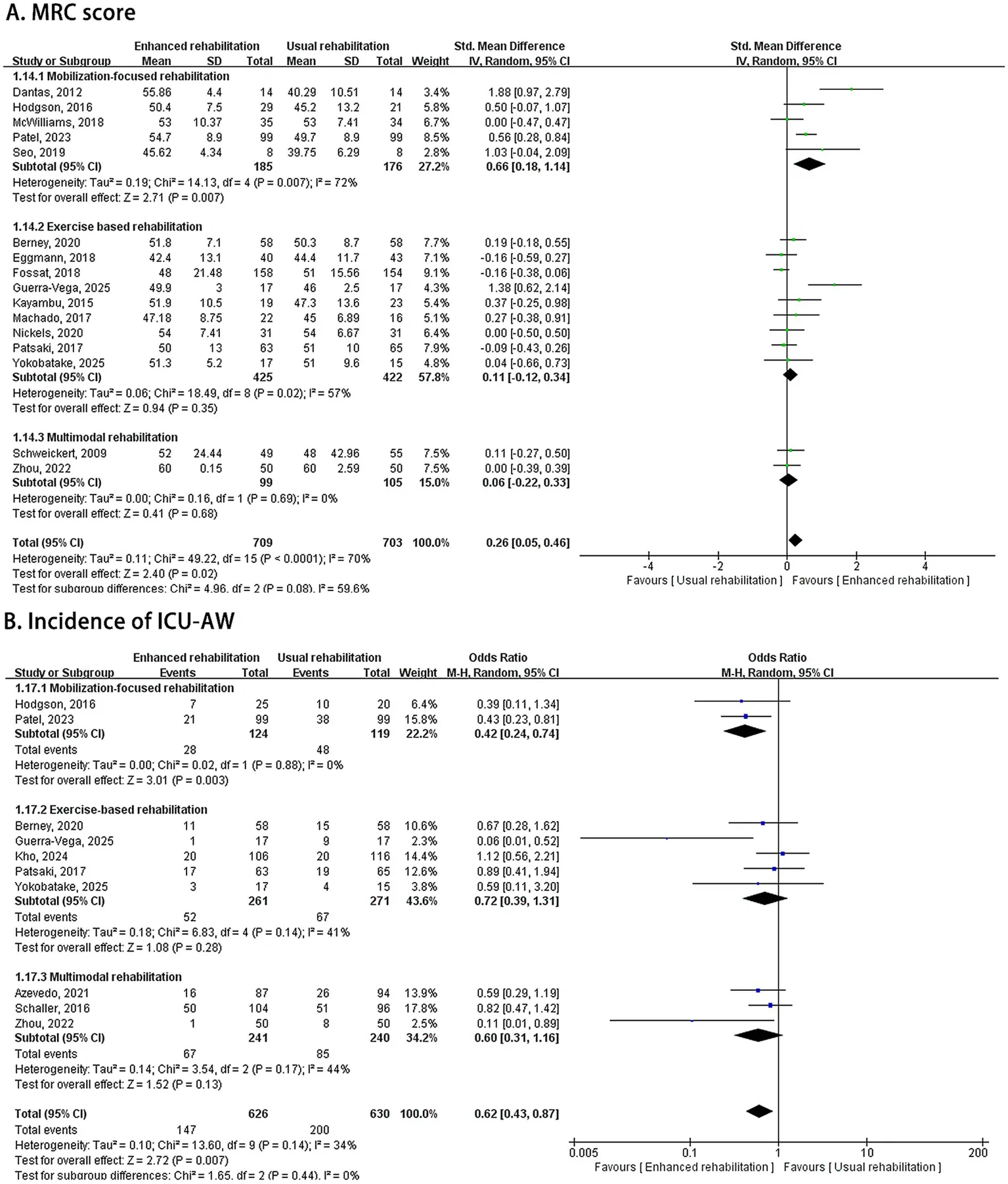
Forest plots showing the effects of physical rehabilitation on muscle strength and ICU-acquired weakness (ICU-AW) compared with usual rehabilitation. **(A)** Medical Research Council (MRC) sum score (primary outcome); **(B)** incidence of ICU-acquired weakness (ICU-AW). Studies were classified according to the dominant rehabilitation strategy (mobilization-focused, exercise-based, or multimodal rehabilitation).

Sixteen studies involving 1,412 participants were included in the primary outcome analysis and were classified according to the dominant rehabilitation strategy as mobilization-focused, exercise-based, or multimodal rehabilitation (Figure 2A). Overall, physical rehabilitation was associated with improved MRC sum score compared with usual rehabilitation (SMD 0.26, 95% CI 0.05–0.46; *p* = 0.02; *I*^2^ = 70%). The effect appeared greater with mobilization-focused rehabilitation (SMD 0.66, 95% CI 0.18–1.14) than with exercise-based (SMD 0.11, 95% CI −0.12 to 0.34) or multimodal rehabilitation (SMD 0.06, 95% CI −0.22 to 0.33), although the test for subgroup differences was not statistically significant (*p* = 0.08). The certainty of evidence was rated as low (Table 2). Funnel plot asymmetry was observed (Supplementary Figure 3) and supported by both Egger’s regression test (*p* = 0.0009) and Begg’s rank correlation test (*p* = 0.015). Heterogeneity exploration using leave-one-out sensitivity analysis, Baujat plots, and influence diagnostics (Supplementary Figures 4, 5) identified the studies by Dantas et al. and Guerra-Vega et al. as the main contributors to between-study heterogeneity. Excluding these studies reduced heterogeneity from 70 to 47%, but the pooled effect was attenuated and no longer statistically significant (SMD 0.13, 95% CI −0.03 to 0.28; *p* = 0.12) (Supplementary Figure 6).

**Table 2:** Summary of findings and GRADE evidence profile for physical rehabilitation in critically ill adults.

Outcome	Studies (participants)	RoB	Inconsistency	Indirectness	Imprecision	Publication bias	GRADE	Effect estimate	Key considerations
MRC sum score (Primary)	16 (1,412)	Not serious	Serious	Not serious	Not serious	Small-study effects suspected	⊕ ⊕ ◯ ◯ Low	SMD 0.26 (0.05–0.46)	Heterogeneity mainly driven by Dantas 2012 and Guerra-Vega 2025; pooled effect was attenuated and no longer statistically significant after leave-one-out.
ICU-acquired weakness	10 (1,256)	Not serious	Not serious	Not serious	Serious	Undetected	⊕ ⊕ ⊕ ◯ Moderate	OR 0.62 (0.43–0.87)	RoB2 no longer downgraded solely for lack of participant blinding.
Handgrip strength	12 (1,162)	Serious	Serious	Not serious	Not serious	Small-study effects suspected	⊕ ◯ ◯ ◯ Very low	SMD 0.02 (−0.18 to 0.23)	Influence largely attributable to Guerra-Vega 2025.
Quadriceps strength	4 (264)	Not serious	Serious	Not serious	Serious	Small-study effects suspected	⊕ ◯ ◯ ◯ Very low	SMD 0.38 (−0.05 to 0.81)	Small evidence base; interpret cautiously.
6-min walk distance	6 (447)	Serious	Serious	Not serious	Serious	Undetected	⊕ ◯ ◯ ◯ Very low	SMD 0.24 (−0.07 to 0.54)	Small evidence base; interpret cautiously.
Functional Independence Measure	3 (401)	Serious	Serious	Not serious	Serious	Small-study effects suspected	⊕ ◯ ◯ ◯ Very low	SMD 0.01 (−0.35 to 0.37)	Very few studies available.
SF-36 physical function domain	13 (1,334)	Serious	Serious	Not serious	Not serious	Small-study effects suspected	⊕ ◯ ◯ ◯ Very low	SMD 0.53 (0.26–0.81)	Heterogeneity reflected differences in follow-up duration, intervention intensity and patient populations rather than a single influential study.
Barthel Index	9 (746)	Not serious	Serious	Not serious	Serious	Small-study effects suspected	⊕ ◯ ◯ ◯ Very low	SMD 0.62 (0.21–1.03)	Several studies contributed moderate heterogeneity.
FSS-ICU	6 (407)	Not serious	Serious	Not serious	Serious	Small-study effects suspected	⊕ ◯ ◯ ◯ Very low	SMD 0.40 (−0.01 to 0.81)	Heterogeneity mainly driven by Guerra-Vega 2025.
Duration of MV	29 (2,809)	Not serious	Serious	Not serious	Not serious	Undetected	⊕ ⊕ ⊕ ◯ Moderate	MD −1.32 (−1.98 to −0.65) days	Several studies contributed moderate heterogeneity.
Overall mortality	27 (4,282)	Serious	Not serious	Not serious	Serious	Undetected	⊕ ⊕ ◯ ◯ Low	OR 0.93 (0.76–1.15)	Consistent neutral effect across studies.
ICU LOS	32 (3,247)	Serious	Not serious	Not serious	Not serious	Undetected	⊕ ⊕ ⊕ ◯ Moderate	MD −1.41 (−2.03 to −0.78) days	Remain robust after leave-one-out analysis.
Hospital LOS	24 (2,767)	Serious	Not serious	Not serious	Serious	Undetected	⊕ ⊕ ◯ ◯ Low	MD −1.29 (−2.59 to −0.01) days	Low heterogeneity (*I*^2^ ≈ 31%); no evidence of publication bias.

Risk of bias was downgraded only when methodological limitations were judged likely to influence the pooled estimate. Lack of participant or personnel blinding alone was not considered sufficient to downgrade objective outcomes. Inconsistency was downgraded when substantial heterogeneity remained unexplained after sensitivity analyses, Baujat plots, and influence diagnostics. Publication bias was downgraded only when visual inspection of funnel plots together with statistical tests for funnel plot asymmetry suggested potential small-study effects.

MV, mechanical ventilation; ICU-AW, ICU-acquired weakness; 6MWD, 6-min walk distance; MRC, Medical Research Council sum score; FSS-ICU, Functional Status Score for the ICU; SF-36 PF, Short Form 36 physical function domain; FIM, Functional Independence Measure; SMD, standardized mean difference; OR, odds ratio; RR, risk ratio; CI, confidence interval.

#### Secondary outcomes: ICU-AW and peripheral muscle strength

Ten studies involving 1,256 participants evaluated the incidence of ICU-AW (Figure 2B). Overall, physical rehabilitation was associated with reduced the incidence of ICU-AW compared with usual rehabilitation (OR 0.62, 95% CI 0.43–0.87; *p* = 0.007; *I*^2^ = 34%). The reduction was most pronounced in mobilization-focused rehabilitation (OR 0.42, 95% CI 0.24–0.74), whereas no significant effects were observed in the exercise-based or multimodal rehabilitation subgroups. However, there was no evidence of subgroup differences (*P* for subgroup difference = 0.44). The certainty of evidence was rated as moderate (Table 2).

**Figure 3:**
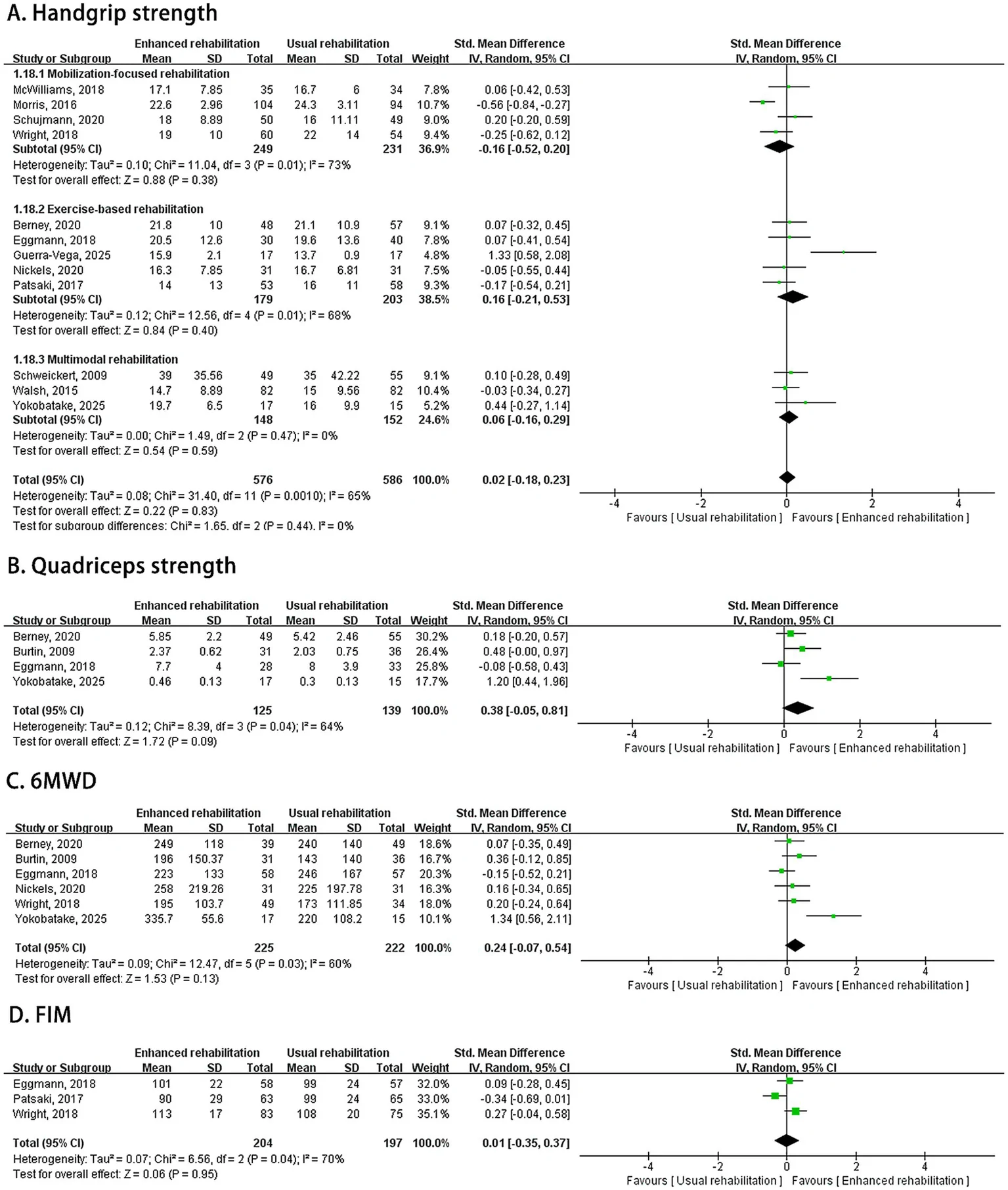
Forest plots showing the effects of physical rehabilitation on peripheral muscle strength and physical function compared with usual rehabilitation. **(A)** Handgrip strength; **(B)** Quadriceps strength; **(C)** 6-min walk distance (6MWD); **(D)** Functional Independence Measure (FIM).

Twelve studies involving 1,162 participants reported handgrip strength (Figure 3A). Physical rehabilitation was not associated with a significant improvement in handgrip strength compared with usual rehabilitation (SMD 0.02, 95% CI −0.18 to 0.23; *p* = 0.83; *I*^2^ = 65%). Funnel plot asymmetry was observed (Supplementary Figure 7) and supported by both Egger’s test (*p* ≤ 0.001) and Begg’s test (*p* = 0.013). Heterogeneity exploration identified Guerra-Vega et al. as the principal contributor to between-study heterogeneity (Supplementary Figures 8, 9). Excluding this study reduced heterogeneity from 65 to 44%, without materially changing the pooled effect estimate (SMD −0.06, 95% CI −0.22 to 0.10; *p* = 0.48) (Supplementary Figure 10). The certainty of evidence was rated as very low (Table 2).

Four studies involving 264 participants assessed quadriceps strength (Figure 3B). Although the pooled estimate favored physical rehabilitation, the difference was not statistically significant (SMD 0.38, 95% CI −0.05 to 0.81; *p* = 0.09; *I*^2^ = 64%). The certainty of evidence was rated as very low (Table 2).

#### Physical function and health-related quality of life

Six studies involving 447 participants assessed the 6-min walk distance (6MWD) (Figure 3C). Physical rehabilitation was not associated with a significant improvement in 6MWD compared with usual rehabilitation (SMD 0.24, 95% CI −0.07 to 0.54; *p* = 0.13; *I*^2^ = 60%). The certainty of evidence was rated as very low (Table 2). Three studies involving 401 participants evaluated the Functional Independence Measure (FIM) (Figure 3D). No significant difference was observed between groups (SMD 0.01, 95% CI −0.35 to 0.37; *p* = 0.95; *I*^2^ = 70%). The certainty of evidence was rated as very low (Table 2).

**Figure 4:**
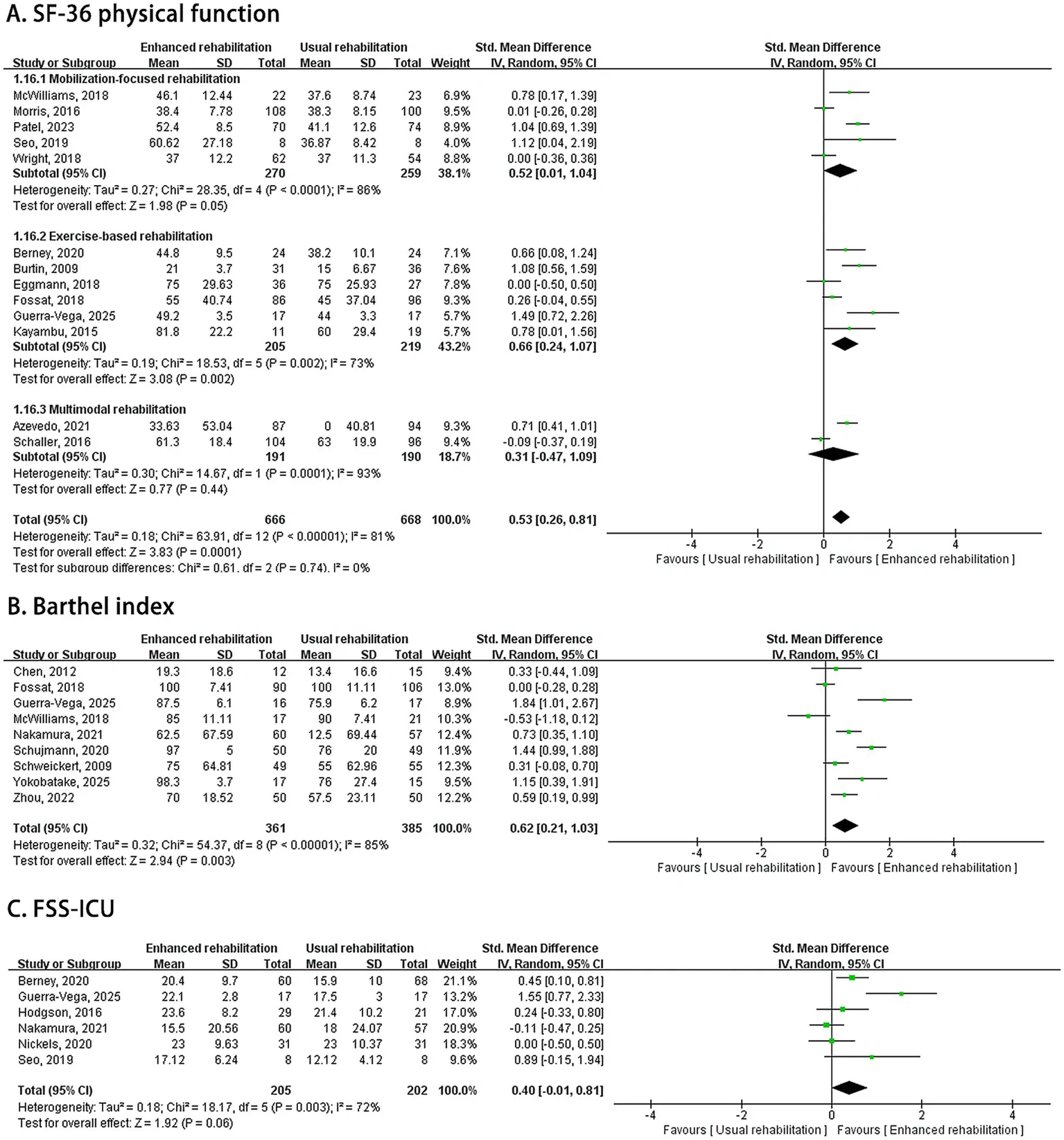
Forest plots showing the effects of physical rehabilitation on health-related quality of life and functional status compared with usual rehabilitation **(A)** SF-36 physical function domain **(B)** Barthel Index **(C)** Functional Status Score for the ICU (FSS-ICU).

Thirteen studies involving 1,334 participants reported the SF-36 physical function domain (Figure 4A). Physical rehabilitation was associated with improved health-related quality of life compared with usual rehabilitation (SMD 0.53, 95% CI 0.26–0.81; *p* < 0.001; *I*^2^ = 81%). Funnel plot asymmetry was observed (Supplementary Figure 11) and supported by Egger’s test (*p* = 0.017), whereas Begg’s test was not statistically significant (*p* = 0.076). Heterogeneity exploration using Baujat plots and influence diagnostics did not identify any single influential study responsible for the observed between-study heterogeneity (Supplementary Figures 12, 13). The certainty of evidence was rated as very low (Table 2). Nine studies involving 746 participants evaluated the Barthel Index (Figure 4B). Physical rehabilitation was associated with improved Barthel Index scores (SMD 0.62, 95% CI 0.21–1.03; *p* = 0.003; *I*^2^ = 85%). Heterogeneity exploration similarly did not identify a single study accounting for the substantial heterogeneity (Supplementary Figures 14, 15). The certainty of evidence was rated as very low (Table 2).

Six studies involving 407 participants assessed the FSS-ICU (Figure 4C). Physical rehabilitation showed a trend toward improved FSS-ICU scores, although the pooled effect did not reach statistical significance (SMD 0.40, 95% CI −0.01 to 0.81; *p* = 0.06; *I*^2^ = 72%). Heterogeneity exploration identified Guerra-Vega et al. as the principal contributor to between-study heterogeneity (Supplementary Figures 16–17). Excluding this study reduced heterogeneity from 72 to 44%, although the pooled effect remained statistically non-significant (SMD 0.20, 95% CI −0.09 to 0.49; *p* = 0.18) (Supplementary Figure 18). The certainty of evidence was rated as very low (Table 2).

### Clinical outcomes

**Figure 5:**
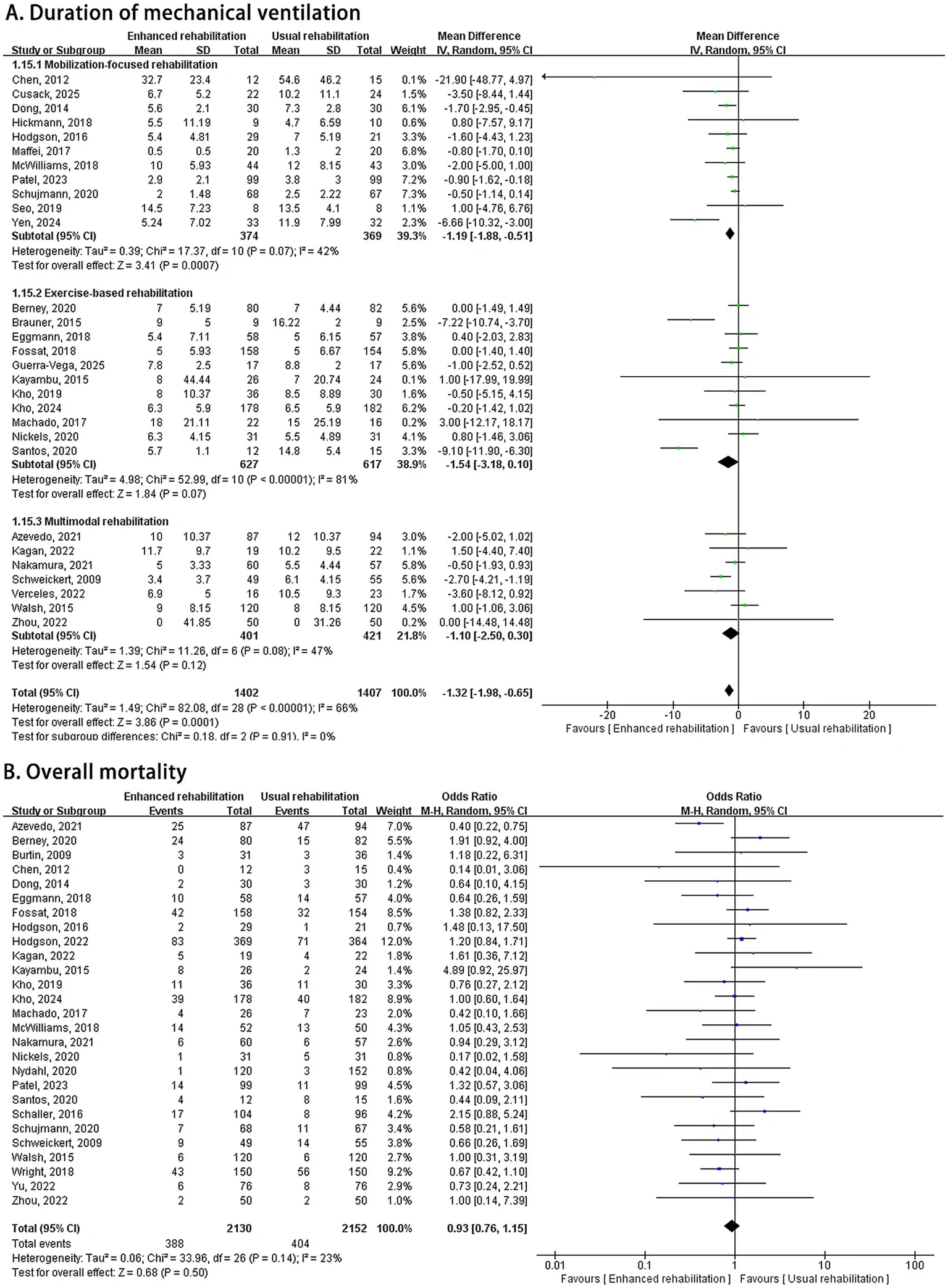
Forest plots showing the effects of physical rehabilitation on clinical outcomes compared with usual rehabilitation. **(A)** Duration of mechanical ventilation. Studies were classified according to the dominant rehabilitation strategy (mobilization-focused, exercise-based, or multimodal rehabilitation); **(B)** Overall mortality.

Twenty-nine studies involving 2,809 participants reported duration of MV (Figure 5A). Overall, physical rehabilitation was associated with reduced MV duration compared with usual rehabilitation (MD −1.32 days, 95% CI −1.98 to −0.65; *p* < 0.001; *I*^2^ = 66%). Significant reductions were observed in the mobilization-focused (MD −1.19 days, 95% CI −1.88 to −0.51), whereas no significant effect was found in exercise-based rehabilitation subgroups (MD −1.54 days, 95% CI −3.18 to 0.10) or in multimodal rehabilitation subgroup (MD −1.10 days, 95% CI −2.50 to 0.30). There was no evidence of subgroup differences (*p* = 0.91). The certainty of evidence was rated as moderate (Table 2). Twenty-seven studies involving 4,282 participants reported overall mortality (Figure 5B). Physical rehabilitation was not associated with a reduction in mortality compared with usual rehabilitation (OR 0.93, 95% CI 0.76–1.15; *p* = 0.50; *I*^2^ = 23%). The certainty of evidence was rated as low (Table 2).

**Figure 6:**
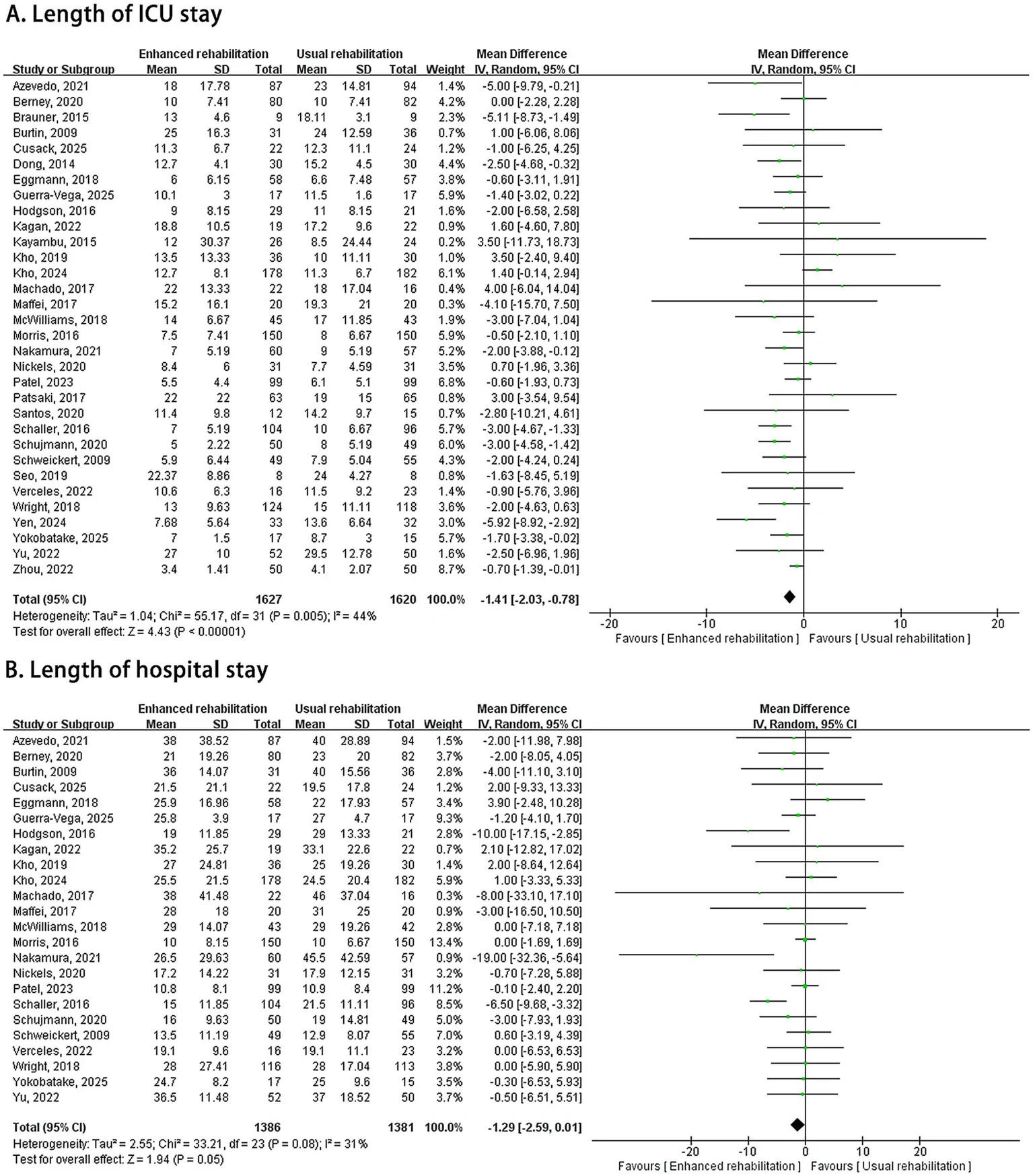
Forest plots showing the effects of physical rehabilitation on length of stay compared with usual rehabilitation. **(A)** ICU length of stay; **(B)** Hospital length of stay.

Thirty-two studies involving 3,247 participants evaluated ICU length of stay (Figure 6A). Physical rehabilitation was associated with reduced ICU length of stay by 1.41 days (MD −1.41 days, 95% CI −2.03 to −0.78; *p* < 0.001; *I*^2^ = 44%). The certainty of evidence was rated as moderate (Table 2). Twenty-four studies involving 2,767 participants assessed hospital length of stay (Figure 6B). Physical rehabilitation showed a trend toward reducing hospital length of stay, although the difference did not reach statistical significance (MD −1.29 days, 95% CI −2.59 to 0.01; *p* = 0.05; *I*^2^ = 31%). The certainty of evidence was rated as low (Table 2). No evidence of publication bias was identified for duration of MV, overall mortality, ICU length of stay, or hospital length of stay (Supplementary Figures 19–22).

## Discussion

This systematic review and meta-analysis synthesized evidence from 40 randomized controlled trials involving 5,135 critically ill adults and provides a comprehensive evaluation of physical rehabilitation across muscle strength, physical function, health-related quality of life, and clinical outcomes. Overall, physical rehabilitation was associated with improved muscle strength, reduced ICU-acquired weakness, enhanced functional recovery, and shorter duration of mechanical ventilation and ICU length of stay, but was not associated with lower mortality.

The improvement in MRC sum score, together with the reduction in ICU-AW incidence, represents the most clinically relevant finding. Both outcomes directly reflect preservation of skeletal muscle function, the principal biological target of physical rehabilitation during critical illness (61). Early mobilization, active exercise, and neuromuscular electrical stimulation may attenuate disuse atrophy, improve neuromuscular activation, and reduce the development of ICU-AW, thereby facilitating subsequent functional recovery (5, 62). Although subgroup analyses suggested a larger effect in mobilization-focused interventions, no statistically significant differences were observed among rehabilitation strategies. Furthermore, sensitivity analyses indicated that the improvement in MRC score was not entirely robust, emphasizing the need for future large, high-quality multicentre trials to confirm the effect of physical rehabilitation on muscle strength.

Functional recovery beyond muscle strength appeared less consistent. Physical rehabilitation significantly improved Barthel Index and SF-36 physical function domain, whereas no significant benefits were observed for 6-min walk distance (6MWD), Functional Independence Measure (FIM), handgrip strength, quadriceps strength, or Functional Status Score for the ICU (FSS-ICU). This pattern probably reflects the multidimensional nature of post-ICU recovery. Measures such as Barthel Index assess overall functional independence and may be more responsive to rehabilitation than isolated assessments of peripheral muscle strength or exercise capacity (63). In contrast, outcomes such as handgrip strength and 6MWD are influenced by multiple physiological and psychological factors, including cardiopulmonary reserve, pre-existing frailty, motivation, and comorbidities, potentially reducing their responses to rehabilitation interventions during critical illness (64, 65).

The observed improvements in MV duration and ICU length of stay suggest that physical rehabilitation may accelerate recovery during the acute phase of critical illness. However, these findings should be interpreted alongside the contemporary evidence base. The two largest multicenter trials, TEAM and CYCLE, reported neutral effects on ventilator-related outcomes despite excellent protocol adherence (16, 17). These apparently discrepant findings likely reflect the progressive incorporation of early mobilization into contemporary usual care (66), thereby reducing the contrast between intervention and control groups compared with earlier trials. Similarly, although rehabilitation was associated with shortened ICU stay, no reduction in mortality was observed. This finding is consistent with previous meta-analyses (9, 14) and suggests that rehabilitation primarily improves recovery among survivors rather than altering the course of the underlying critical illness, which remains largely determined by disease severity and organ failure.

A major strength of this review is the exploration of heterogeneity and publication bias. Heterogeneity analyses indicated that between-study variability was primarily attributable to differences in rehabilitation protocols, intervention intensity, patient populations, and outcome assessment methods, although one or two small studies disproportionately influenced several outcomes. Despite this variability, no significant interactions were observed across rehabilitation strategies, supporting the overall consistency of treatment effects.

This review has several limitations. First, considerable clinical heterogeneity existed across studies with respect to rehabilitation modality, timing, intensity, duration, and patient characteristics, limiting the ability to identify the optimal rehabilitation strategy. Second, several functional outcomes were supported by relatively few studies, resulting in low or very low certainty of evidence. Third, the majority of included studies are single-center trials conducted in high-resource ICU settings with dedicated physiotherapy staffing, which may limit the external validity and generalizability of findings to resource-limited settings. Finally, long-term outcomes beyond 6 months were infrequently reported, precluding robust assessment of the durability of rehabilitation benefits.

## Conclusion

Physical rehabilitation was associated with improved muscle strength, reduced ICU-acquired weakness, enhanced functional recovery, and shorter duration of mechanical ventilation and ICU length of stay in critically ill adults, but not with lower mortality. Further high-quality multicenter randomized controlled trials are needed to confirm these findings and define the optimal rehabilitation strategy.

## Data Availability

The original contributions presented in the study are included in the article/Supplementary material, further inquiries can be directed to the corresponding author.
