# Supplementary material for: Systematic review and meta-analysis of physical rehabilitation on functional recovery in critically ill patients
Source: Front Med (Lausanne). 2026 Aug 18;13:1877465. doi: 10.3389/fmed.2026.1877465 (PMC13529707; doi:10.3389/fmed.2026.1877465)
Supplement: Supplementary file 1 [file Data_Sheet_1.docx]

**SUPPLEMENTARY APPENDIX**

**Physical rehabilitation for functional recovery in critically ill adults: a systematic review and meta-analysis**

This systematic review and meta-analysis was registered in PROSPERO (CRD42023462649).

**Contents**

[**Supplementary figure 1.** Risk-of-bias assessment of individual randomized controlled trials using the Cochrane Risk of Bias 2 (RoB 2) tool.	3](#_Toc234096876)

[**Supplementary figure 2.** Summary of risk-of-bias judgments across all included randomized controlled trials according to the Cochrane Risk of Bias 2 (RoB 2) tool.	4](#_Toc234096877)

[**Supplementary Figure 3. Funnel plot for the primary outcome (MRC sum score).**	5](#_Toc234096878)

[**Supplementary Figure 4. Baujat plot for the primary outcome (MRC sum score).**	6](#_Toc234096879)

[**Supplementary Figure 5. Influence diagnostics for the primary outcome (MRC sum score).**	7](#_Toc234096880)

[**Supplementary Figure 6. Sensitivity analysis of the primary outcome (MRC sum score).**	8](#_Toc234096881)

[**Supplementary Figure 7. Funnel plot for handgrip strength.**	9](#_Toc234096882)

[**Supplementary Figure 8. Baujat plot for handgrip strength.**	10](#_Toc234096883)

[**Supplementary Figure 9. Influence diagnostics for handgrip strength.**	11](#_Toc234096884)

[**Supplementary Figure 10. Sensitivity analysis of handgrip strength.**	12](#_Toc234096885)

[**Supplementary Figure 11. Funnel plot for the SF-36 physical function domain.**	13](#_Toc234096886)

[**Supplementary Figure 12. Baujat plot for the SF-36 physical function domain.**	14](#_Toc234096887)

[**Supplementary Figure 13. Influence diagnostics for the SF-36 physical function domain.**	15](#_Toc234096888)

[**Supplementary Figure 14. Baujat plot for the Barthel Index.**	16](#_Toc234096889)

[**Supplementary Figure 15. Influence diagnostics for the Barthel Index.**	17](#_Toc234096890)

[**Supplementary Figure 16. Baujat plot for the Functional Status Score for the ICU (FSS-ICU).**	18](#_Toc234096891)

[**Supplementary Figure 17. Influence diagnostics for the Functional Status Score for the ICU (FSS-ICU).**	19](#_Toc234096892)

[**Supplementary Figure 18. Sensitivity analysis of the Functional Status Score for the ICU (FSS-ICU).**	20](#_Toc234096893)

[**Supplementary Figure 19. Funnel plot for duration of mechanical ventilation.**	21](#_Toc234096894)

[**Supplementary Figure 20. Funnel plot for overall mortality.**	22](#_Toc234096895)

[**Supplementary Figure 21. Funnel plot for ICU length of stay.**	23](#_Toc234096896)

[**Supplementary Figure 22. Funnel plot for hospital length of stay.**	24](#_Toc234096897)

# **Supplementary figure 1.** Risk-of-bias assessment of individual randomized controlled trials using the Cochrane Risk of Bias 2 (RoB 2) tool.

# **Supplementary figure 2.** Summary of risk-of-bias judgments across all included randomized controlled trials according to the Cochrane Risk of Bias 2 (RoB 2) tool.

# **Supplementary Figure 3. Funnel plot for the primary outcome (MRC sum score).**

Funnel plot assessing potential publication bias for studies reporting the Medical Research Council (MRC) sum score.

# **Supplementary Figure 4. Baujat plot for the primary outcome (MRC sum score).**

Baujat plot identifying the contribution of individual studies to overall heterogeneity and their influence on the pooled effect estimate for the Medical Research Council (MRC) sum score. Study #2 represents the study by Dantas et al. and study #5 represents the study by Guerra-Vega et al.

# **Supplementary Figure 5. Influence diagnostics for the primary outcome (MRC sum score).**

Influence diagnostics showing the impact of individual studies on the pooled effect estimate and heterogeneity for the Medical Research Council (MRC) sum score. Diagnostic measures include externally standardized residuals, DFFITS, Cook's distance, covariance ratio, leave-one-out heterogeneity estimates, hat values, and study weights.

# **Supplementary Figure 6. Sensitivity analysis of the primary outcome (MRC sum score).**

Forest plot showing the sensitivity analysis after exclusion of the influential studies identified by the heterogeneity analyses (Dantas et al. and Guerra-Vega et al.), demonstrating the corresponding changes in the pooled effect estimate and statistical heterogeneity.

# **Supplementary Figure 7. Funnel plot for handgrip strength.**

Funnel plot assessing potential publication bias for studies reporting handgrip strength.

# **Supplementary Figure 8. Baujat plot for handgrip strength.**

Baujat plot illustrating the contribution of individual studies to overall heterogeneity and their influence on the pooled effect estimate for handgrip strength. Study #3 represents the study by Guerra-Vega et al.

# **Supplementary Figure 9. Influence diagnostics for handgrip strength.**

Influence diagnostics showing the impact of individual studies on the pooled effect estimate and between-study heterogeneity for handgrip strength. Diagnostic measures include externally standardized residuals, DFFITS, Cook's distance, covariance ratio, leave-one-out heterogeneity estimates, hat values, and study weights.

# **Supplementary Figure 10. Sensitivity analysis of handgrip strength.**

Forest plot showing the sensitivity analysis after exclusion of the influential study identified by the heterogeneity analyses (Guerra-Vega et al.), demonstrating the corresponding changes in the pooled effect estimate and statistical heterogeneity.

# **Supplementary Figure 11. Funnel plot for the SF-36 physical function domain.**

Funnel plot assessing potential publication bias for studies reporting the SF-36 physical function domain.

# **Supplementary Figure 12. Baujat plot for the SF-36 physical function domain.**

Baujat plot illustrating the contribution of individual studies to overall heterogeneity and their influence on the pooled effect estimate for the SF-36 physical function domain.

# **Supplementary Figure 13. Influence diagnostics for the SF-36 physical function domain.**

Influence diagnostics showing the impact of individual studies on the pooled effect estimate and between-study heterogeneity for the SF-36 physical function domain. Diagnostic measures include externally standardized residuals, DFFITS, Cook's distance, covariance ratio, leave-one-out heterogeneity estimates, hat values, and study weights.

# **Supplementary Figure 14. Baujat plot for the Barthel Index.**

Baujat plot illustrating the contribution of individual studies to overall heterogeneity and their influence on the pooled effect estimate for the Barthel Index.

# **Supplementary Figure 15. Influence diagnostics for the Barthel Index.**

Influence diagnostics showing the impact of individual studies on the pooled effect estimate and between-study heterogeneity for the Barthel Index. Diagnostic measures include externally standardized residuals, DFFITS, Cook's distance, covariance ratio, leave-one-out heterogeneity estimates, hat values, and study weights.

# **Supplementary Figure 16. Baujat plot for the Functional Status Score for the ICU (FSS-ICU).**

Baujat plot illustrating the contribution of individual studies to overall heterogeneity and their influence on the pooled effect estimate for the Functional Status Score for the ICU (FSS-ICU). Study #2 represents the study by Guerra-Vega et al.

# **Supplementary Figure 17. Influence diagnostics for the Functional Status Score for the ICU (FSS-ICU).**

Influence diagnostics showing the impact of individual studies on the pooled effect estimate and between-study heterogeneity for the Functional Status Score for the ICU (FSS-ICU). Diagnostic measures include externally standardized residuals, DFFITS, Cook's distance, covariance ratio, leave-one-out heterogeneity estimates, hat values, and study weights.

# **Supplementary Figure 18. Sensitivity analysis of the Functional Status Score for the ICU (FSS-ICU).**

Forest plot showing the sensitivity analysis after exclusion of the influential study identified by the heterogeneity analyses (Guerra-Vega et al.), demonstrating the corresponding changes in the pooled effect estimate and statistical heterogeneity.

# **Supplementary Figure 19. Funnel plot for duration of mechanical ventilation.**

Funnel plot assessing potential publication bias for studies reporting duration of mechanical ventilation.

# **Supplementary Figure 20. Funnel plot for overall mortality.**

Funnel plot assessing potential publication bias for studies reporting mortality.

# **Supplementary Figure 21. Funnel plot for ICU length of stay.**

Funnel plot assessing potential publication bias for studies reporting ICU length of stay.

# **Supplementary Figure 22. Funnel plot for hospital length of stay.**

Funnel plot assessing potential publication bias for studies reporting hospital length of stay.
